# Intrahepatic Cholangiocarcinoma Masquerading as Liver Abscess: A Case Report With Multimodal Imaging

**DOI:** 10.1002/ccr3.71615

**Published:** 2025-12-03

**Authors:** Chunxiao Song, Yang Xu, Yuanyuan Zhang, Runfeng Miao

**Affiliations:** ^1^ Emergency Department The Affiliated Hospital of Yangzhou University, Yangzhou University Yangzhou China; ^2^ Emergency Department The First Affiliated Hospital of Soochow University Suzhou China

**Keywords:** case report, intrahepatic cholangiocarcinoma (ICC), liver abscess, misdiagnosis, multimodal imaging

## Abstract

The presentation of intrahepatic cholangiocarcinoma (ICC) often masquerades as a pyogenic liver abscess. Therefore, atypical imaging features on multimodal studies, together with markedly elevated levels of Gamma‐glutamyl Transpeptidase (GGT), Alkaline Phosphatase (ALP) and Carbohydrate antigen 19‐9 (CA19‐9), must alert clinicians to the possibility of underlying malignancy to prevent diagnostic delay.

## 1 Introduction

Intrahepatic cholangiocarcinoma (ICC) is the second most common primary malignant liver tumor after hepatocellular carcinoma, accounting for approximately 10%–15% of primary liver cancers, with a globally increasing incidence [1]. Known risk factors include primary sclerosing cholangitis, liver fluke infestation, hepatolithiasis, biliary developmental anomalies, viral hepatitis (especially chronic hepatitis B and C), choledochal cysts, and diabetes mellitus [2, 3]. Notably, intrahepatic stones are not only a significant high‐risk factor for ICC but also a primary infectious pathway of pyogenic liver abscesses. Early clinical presentation of ICC is often insidious. Coexisting infection frequently leads to delayed diagnosis or misdiagnosis, significantly impacting treatment strategy formulation and patient prognosis [4]. Although radical surgical resection remains the treatment of choice, the postoperative five‐year survival rate for patients remains suboptimal [5]. The combined application of multimodal imaging examinations (such as ultrasound, computed tomography [CT], magnetic resonance imaging [MRI]) has significantly improved the diagnostic accuracy of ICC, providing valuable time for early identification and intervention [6]. This article reports a case of a patient presenting with chills and fever as initial symptoms, who exhibited multiple biliary ductal dilatations and stones, initially diagnosed as a pyogenic liver abscess. Subsequent systematic imaging evaluation ultimately confirmed ICC, facilitating timely surgical intervention.

## 2 Case History

**FIGURE 1:**
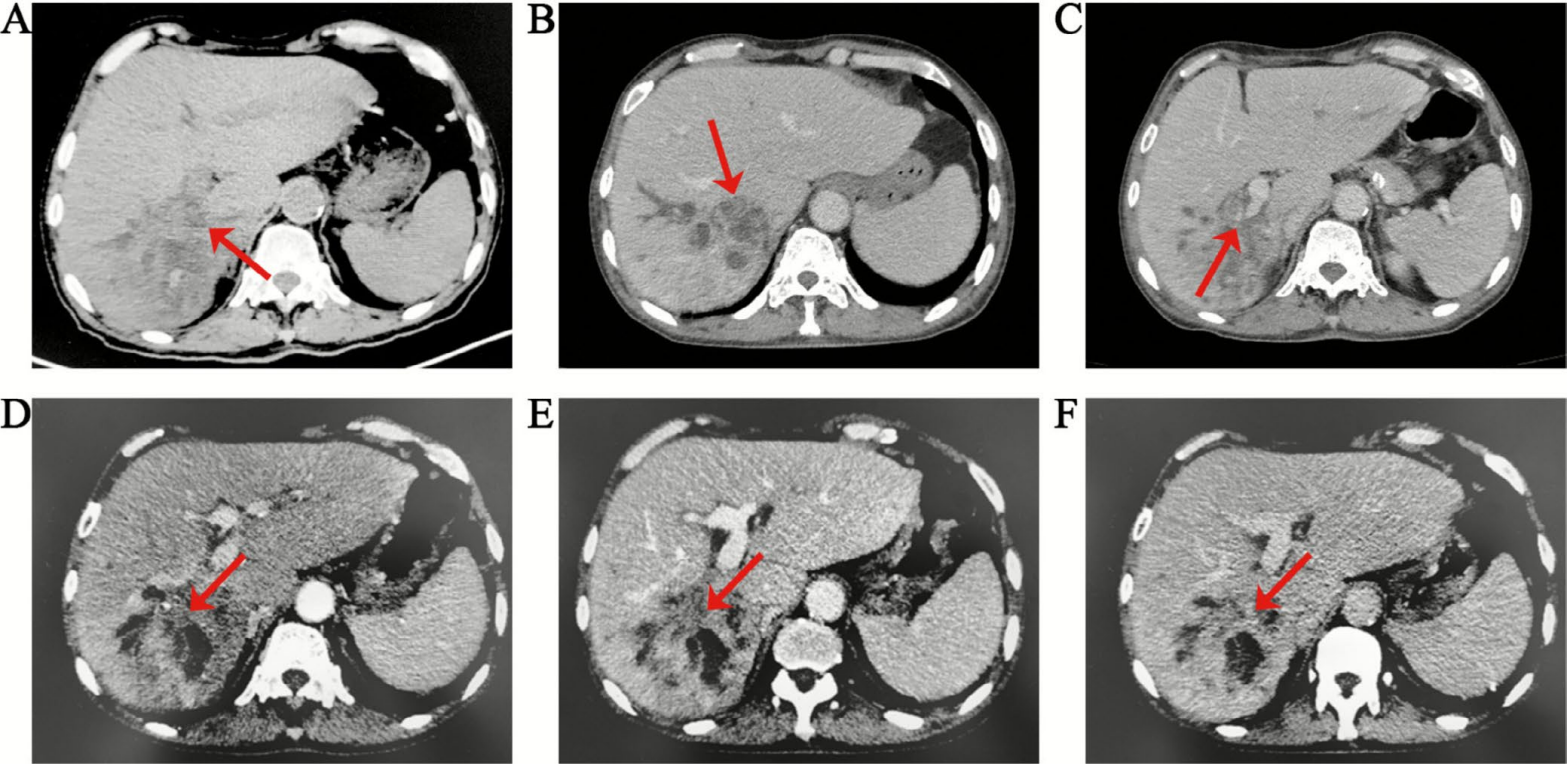
Computed tomography (CT) and contrast enhanced CT. (A) On plain CT scan, the density of liver parenchyma was slightly reduced; (B) Enhanced CT showed a honeycomb‐like low‐density shadow in the right sector (segments V to VIII) near the hepatic hilum; (C) Enhanced CT showed that the intrahepatic segment of the right portal vein was nearly occluded; (D–F) Enhanced CT showed thickening of the intrahepatic bile duct wall in the right lobe near the hepatic hilum with mild delayed enhancement (D: Arterial phase, E: Venous phase, F: Delayed phase).

A 67‐year‐old male patient was admitted to the emergency department on March 12, 2025, due to “chills and fever for one day.” One day prior to admission, he experienced sudden‐onset chills and fever (temperature not measured), accompanied by profuse sweating and one episode of vomiting gastric contents, without receiving any specific treatment. On the afternoon of March 12, the patient again experienced chills and rigor. He presented to a community health service station, where the temperature of 38.5°C was recorded. He received intravenous infusion of cefuroxime and lysine acetylsalicylate, but the fever did not subside. He was then transferred to the emergency department of our hospital, where repeat temperature measurement showed 39.3°C. Emergency laboratory tests revealed elevated white blood cell count (WBC 12.40 × 10^9^/L), neutrophil count (11.86 × 10^9^/L), and high‐sensitivity C‐reactive protein (hs‐CRP 129.02 mg/L), indicating an inflammatory response. Albumin (ALB 34.3 g/L), total bilirubin (TBIL 12.3 μmol/L), alanine aminotransferase (ALT 23 U/L), aspartate aminotransferase (AST 26 U/L), gamma‐glutamyl transpeptidase (GGT 287 U/L), and alkaline phosphatase (ALP 398 U/L) were assessed. Elevated GGT and ALP levels suggest biliary obstruction or bile stasis. Non‐contrast computed tomography (CT) scan of the entire abdomen demonstrated: Multiple stones are present in the bile ducts within segments V to VIII of the right liver sector, with decreased density of the surrounding liver parenchyma; Status post cholecystectomy; Dilatation of intra‐ and extrahepatic bile ducts (Figure 1A). He was admitted to the hospital with a preliminary diagnosis of infectious fever.

The patient's past health status was fair. He had a medical history of hypertension and type 2 diabetes mellitus. More than 20 years ago, he underwent cholecystectomy at another hospital for gallstones; details are unavailable. He had a history of long‐term smoking and alcohol consumption. He denied any history of hepatitis (including hepatitis B or C) or close contact history, parasitic infection, or family history of genetic diseases.

### 2.1 Admission Physical Examination

Axillary temperature 39.3°C. The Glasgow Coma Scale (GCS) score was 15/15, with no jaundice observed; the sclerae were anicteric. Cardiopulmonary auscultation showed no significant abnormalities. The abdomen was flat and soft, with mild tenderness in the right upper quadrant. No muscle guarding or rebound tenderness was noted. Murphy's sign was negative. The liver and spleen were not palpable below the costal margin. Percussion tenderness over the liver area was positive. Shifting dullness was negative. Bowel sounds were normal (approximately 4 times per minute). Muscle strength and tone in all four limbs were normal. Pathological reflexes were not elicited.

## 3 Investigations and Treatment

Post‐admission blood culture revealed infection with *Escherichia coli* . Based on the clinical presentation and initial non‐contrast CT findings, a preliminary diagnosis of an infectious fever, with pyogenic liver abscess as a leading consideration, was made. Meropenem was administered for anti‐infective therapy.

To characterize the hypodense hepatic lesions, contrast‐enhanced CT of the entire abdomen was performed on March 15. Imaging findings (Figure 1B–F): A honeycomb‐pattern hypodense lesion (approx. 4.2 cm × 3.1 cm) was observed in the right (segments V to VIII) sector near the hepatic hilum, consistent with the classic “honeycomb sign” of liver abscess. Multiple intrahepatic bile duct stones with biliary dilatation were noted in the right sector (segments V to VIII). Adjacent to the abscess (right lobe near the hilum), the intrahepatic bile duct walls showed thickening with mild delayed enhancement, raising suspicion for possible cholangiocarcinoma. Luminal occlusion was noted in the proximal intrahepatic segment of the right portal vein, suggestive of secondary changes requiring differentiation between thrombophlebitis and tumor involvement. Post‐cholecystectomy changes were present, accompanied by common bile duct dilatation.

**FIGURE 2:**
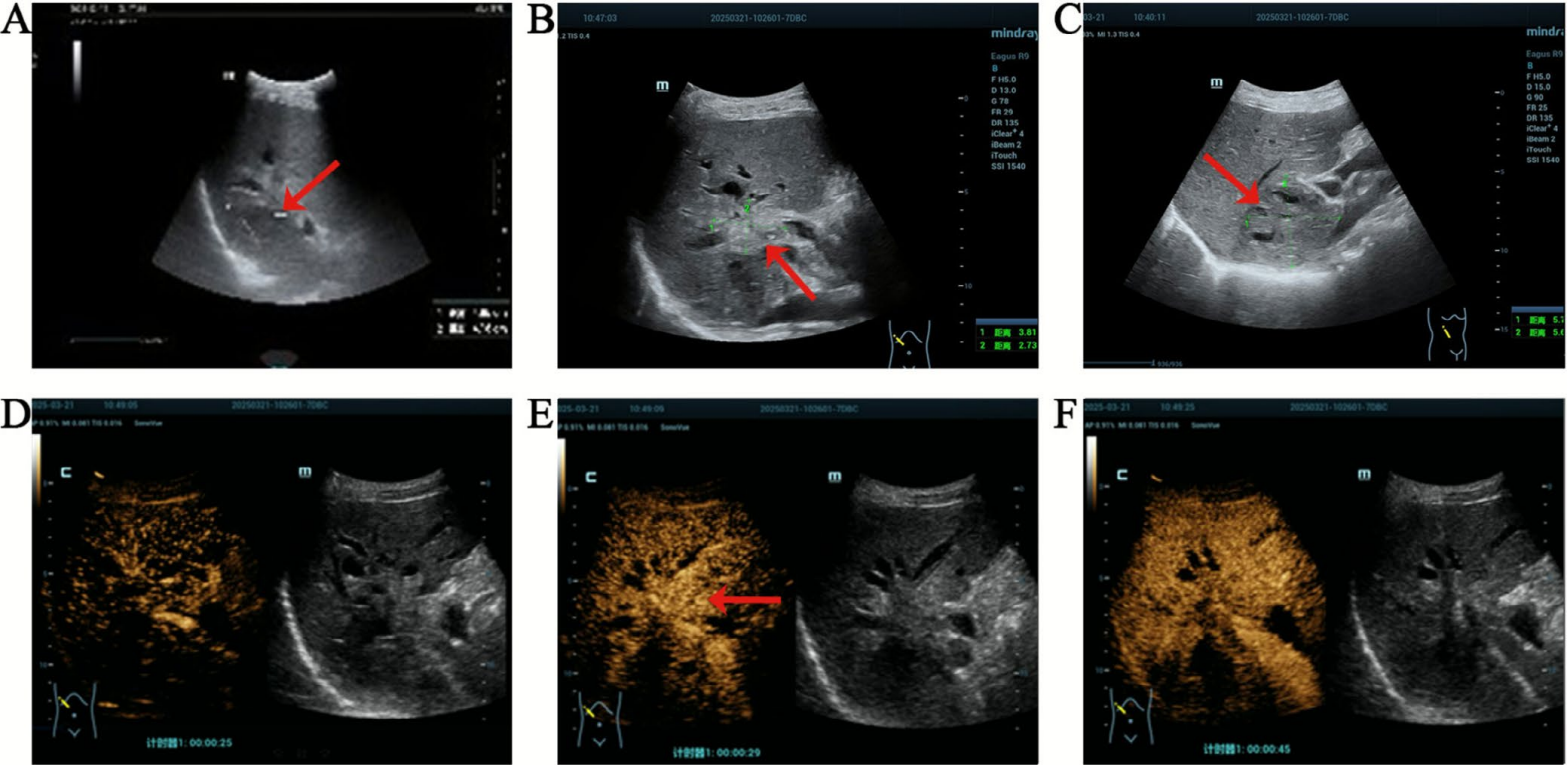
Ultrasound and contrast‐enhanced ultrasound (CEUS). (A) Routine ultrasound on March 16 showed patchy hypoechoic areas in the right lobe of the liver; (B) Ultrasound reexamination on March 21 showed a slightly hyperechoic mass at the hepatic hilum. (C) In the right sector (segments V to VIII) of the liver, there was a solid predominantly cystic and solid mixed echo area near the dome of the diaphragm. (D) CEUS, liver parenchyma imaging began at 22 s; (E) The enhancement peaked at 29 s; (F) Overall hypoenhancement of the mass for 45 s.

Abdominal ultrasound re‐examination on March 16 revealed a patchy hypoechoic area (approx. 42 mm × 40 mm) in the right hepatic lobe, with ill‐defined margins, posterior acoustic enhancement, and scattered punctate hyperechoic foci internally. Color Doppler flow imaging (CDFI) demonstrated relatively abundant blood flow signals within the surrounding parenchyma (Figure 2A). Ultrasound findings suggested an abscess without significant liquefaction. Given the absence of definite liquefied areas or septations within the lesion (indicating early‐stage abscess), ultrasound‐guided percutaneous drainage was deferred. Anti‐infective therapy was continued with close monitoring.

Despite the initial presentation and imaging features suggestive of a liver abscess, the absence of significant liquefaction on follow‐up imaging, and the persistent irregular rim enhancement on contrast‐enhanced studies were atypical for a simple pyogenic abscess and heightened our suspicion for an underlying malignancy such as ICC. Repeat abdominal ultrasound on March 21 identified a mildly hyperechoic mass (approx. 38 × 27 mm) near the hepatic hilum. CDFI showed the mass encircling the hepatic artery, with several dilated intrahepatic bile ducts adjacent to it. Some bile ducts contained hyperechoic foci with acoustic shadows and poor sound transmission (Figure 2B). A mixed solid‐cystic echoic lesion (predominantly solid, approx. 57 × 57 mm) was observed in the right sector (segments V to VIII) near the diaphragmatic dome (Figure 2C). The right hepatic space‐occupying lesion was highly suspicious for ICC. To characterize the hemodynamic features and improve diagnostic specificity, contrast‐enhanced ultrasound (CEUS) was performed (Figure 2D–F). A 2.4 mL bolus of sulfur hexafluoride microbubble contrast agent (SonoVue) was administered via the left median cubital vein. In the arterial phase (peak enhancement at 29 s post‐injection), the hilar mass exhibited rapid homogeneous hyperenhancement, demonstrating a “fast‐in and fast‐out” pattern. In the delayed phase, the mass showed homogeneous hypoenhancement with well‐defined margins, measuring enlarged dimensions of 53 × 31 mm.

**FIGURE 3:**
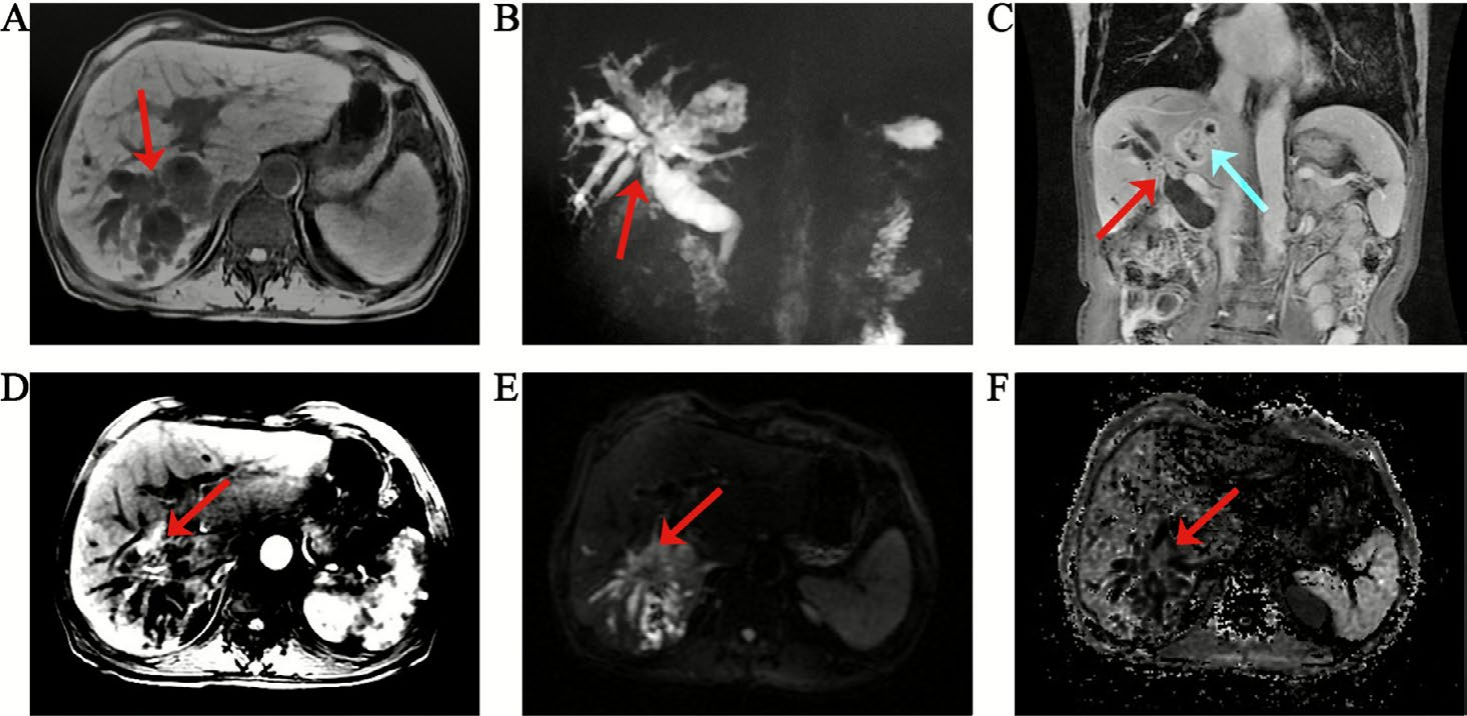
Magnetic resonance imaging (MRI), magnetic resonance cholangiopancreatography (MRCP), and contrast enhanced MRI. (A) MRI T2‐weighted image, honeycomb‐like mixed signal shadow was found in the right sector (segments V to VIII) near the hepatic hilum, and patchy high signal shadow was seen at the edge. (B) MRCP, the intrahepatic bile ducts of the right sector (segments V to VIII) were dilated to varying degrees with multiple filling defects; (C) MRI Enhanced coronal plane, red arrow: Hilar bile duct stenosis and thickening, blue arrow: Honeycomb‐like mixed signal shadow in the right sector (segments V to VIII) of the liver; (D) T2‐weighted image, patchy high signal shadow at the edge of the right sector (segments V to VIII) of the liver; (E) DWI, slightly high signal; (F) ADC, no significant decrease was observed.

Laboratory tests revealed significantly elevated serum CA19‐9 (1547 U/mL), while alpha‐fetoprotein (AFP) and carcinoembryonic antigen (CEA) levels were within normal ranges. Combined with imaging characteristics, a clinical preliminary diagnosis of ICC was established, though definitive confirmation required histopathological results. Given that surgical resection is the primary curative approach for ICC, preoperative systematic assessment of biliary involvement extent, lesion invasion depth, and intrahepatic metastasis was necessary. The patient underwent abdominal MRI, magnetic resonance cholangiopancreatography (MRCP), and contrast‐enhanced MRI on March 25 and 28, with the following imaging features (Figure 3A–F): A honeycomb‐pattern mixed‐signal lesion (5.3 × 4.1 cm) with ill‐defined margins was observed near the hepatic hilum in the right sector (segments V to VIII), containing multiple cystic long T2 signals; localized stenosis with wall thickening was observed in the right sector (segments V to VIII) bile ducts, containing multifocal punctate and patchy short T1/T2 signals (suggestive of stones or debris) and associated with proximal biliary dilatation; near‐occlusion of the intrahepatic segment of the right portal vein; diffusion‐weighted imaging (DWI) showed slightly hyperintense signals without significant reduction in apparent diffusion coefficient (ADC) values; contrast enhancement demonstrated peripheral and septal enhancement without cystic area enhancement; small lymph nodes were noted in the hepatic hilar region, with no enlarged para‐aortic lymph nodes.

**FIGURE 4:**
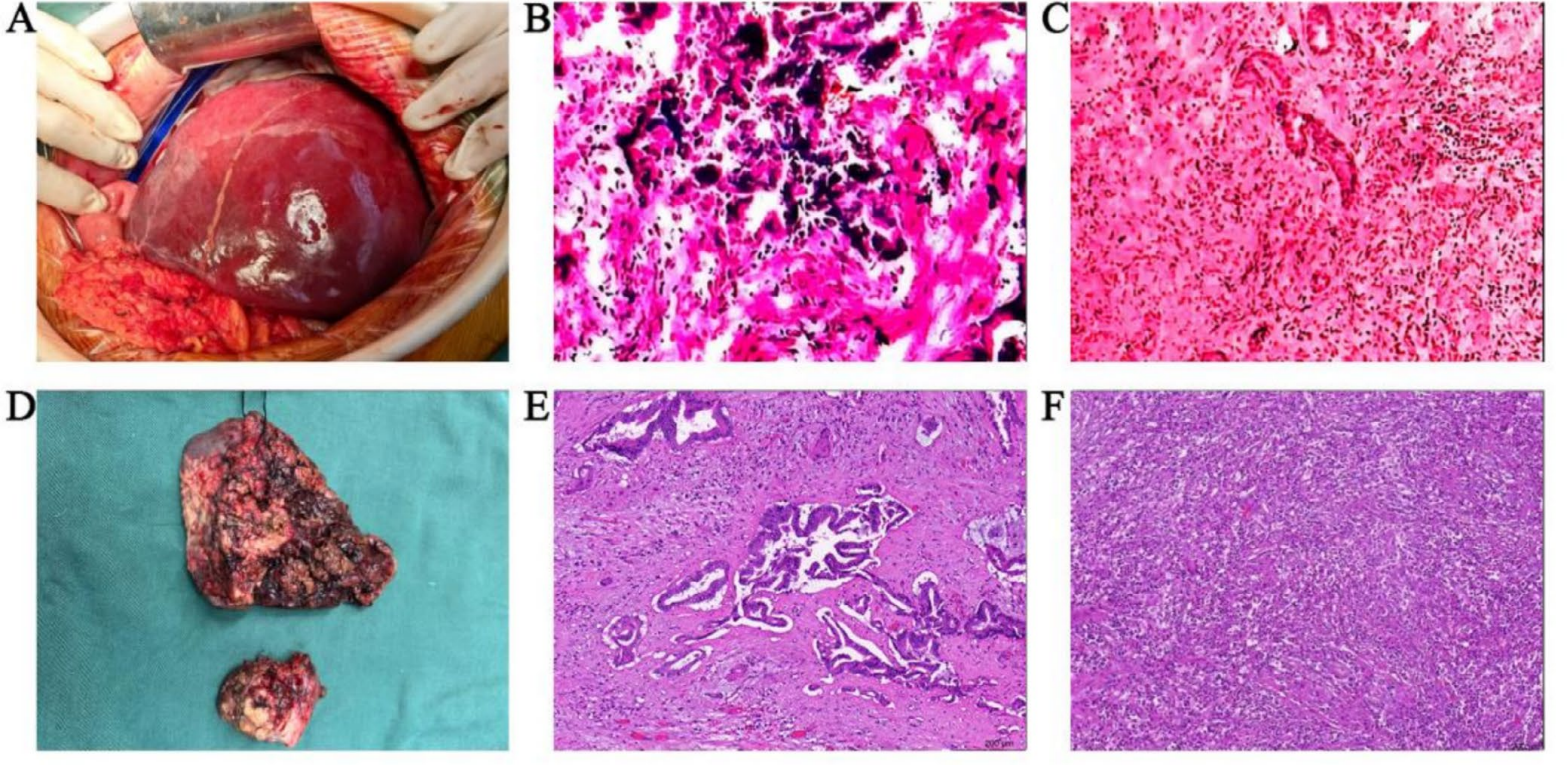
Surgically resected liver tissue and pathology. (A) Intraoperative exposure of the liver; a nodule on the surface of segment VIII was identified. (B) Frozen‐section analysis of the biliary margin was positive, showing high‐grade dysplasia of the biliary epithelium. (C) Intraoperative rapid pathology in the caudate lobe of the liver suggested moderately differentiated adenocarcinoma. (D) Resection of the right liver sector (segments V to VIII) and partial caudate. (E) Right liver pathology: well to moderately differentiated intrahepatic cholangiocarcinoma. The tumor microenvironment showed significant neutrophilic infiltration, consistent with tumor‐associated suppurative inflammation. (F) Pathology of the caudate lobe: diffuse hyperplasia with degeneration of fibrous tissue, a large number of acute and chronic inflammatory cell infiltration.

After antibiotic treatment, the patient experienced no further fever episodes and showed a decline in blood cell counts and inflammatory markers compared to previous levels. Following completion of the preoperative evaluation and exclusion of contraindications, the patient underwent radical right hepatectomy including resection of the caudate lobe tumor, as well as common bile duct exploration with T‐tube drainage on April 8, 2025. Intraoperative findings: Upon exploration, a nodule on the surface of segment VIII was identified (Figure 4A). Its excision and subsequent frozen‐section examination confirmed nodular hyperplasia and ruled out tumor infiltration. Frozen‐section analysis of the biliary margin revealed a positive margin, showing high‐grade dysplasia of the biliary epithelium (Figure 4B). Frozen section of the caudate lobe lesion suggested moderately differentiated adenocarcinoma (likely biliary origin) (Figure 4C), leading to right liver sector (segments V to VIII) and partial caudate lobectomy (Figure 4D). Postoperative pathology confirmed intrahepatic cholangiocarcinoma (well‐to‐moderately differentiated) in the right hemi‐liver lesion, with associated infection and perineural invasion (Figure 4E). The caudate lobe lesion was identified as a hepatic abscess (fibroplasia with acute and chronic inflammation) (Figure 4F). The immunohistochemical profile showed positivity for CK7, CK19, CK8, and CK18, supporting biliary epithelial origin. HER2 was weakly positive (1+). S‐100 positivity confirmed neural invasion. The mismatch repair proteins MLH1, PMS2, MSH2, and MSH6 showed positive expression.

## 4 Discussion

This case highlights the complexity in differentiating intrahepatic cholangiocarcinoma (ICC) from liver abscess (LA) and their therapeutic management, particularly when clinical or imaging features coexist. ICC typically presents with insidious onset and nonspecific symptoms, with jaundice and weight loss being common manifestations, whereas LA predominantly manifests with fever as the cardinal clinical feature. Upon initial presentation, this patient exhibited fever, leukocytosis, and a “honeycomb‐pattern” hypodense lesion on imaging, consistent with classic signs of pyogenic liver abscess. Previous studies indicate that approximately 12.5% of ICC cases may present with fever, and overlapping symptoms frequently lead to misdiagnosis as infectious diseases [7].

Regarding imaging diagnosis, ICC exhibits characteristic enhancement patterns on contrast‐enhanced CT: central hypoattenuation during arterial and portal venous phases due to abundant fibrous stroma, with marked delayed‐phase hyperenhancement. MRI diffusion‐weighted imaging (DWI) enhances ICC detection sensitivity, with ADC values typically significantly lower than adjacent normal liver parenchyma [8]. Combining DWI with hepatobiliary phase imaging improves differentiation from LA by highlighting ICC's hypointense margins and diffusion restriction features [9]. This patient's abdominal contrast‐enhanced CT revealed mild delayed enhancement in the right sector (segments V to VIII) bile ducts near the hilum—an atypical presentation, easily leading to a missed diagnosis. Fortunately, contrast‐enhanced ultrasound demonstrated a “fast‐in and fast‐out” enhancement pattern in the hilar mass, highly suggestive of malignancy. Additionally, MRCP and contrast‐enhanced MRI showed intrahepatic biliary dilatation with multiple filling defects in the right sector (segments V to VIII), but DWI revealed only mild hyperintensity without significant ADC reduction—deviating from typical ICC manifestations.

Among laboratory markers, C‐reactive protein (CRP) serves as an independent predictor for distinguishing LA from ICC and holds the highest diagnostic value among inflammatory indicators [7], yet its discriminative capacity remains limited for ICC with concurrent infection. CA19‐9, the most utilized tumor marker for ICC, demonstrates validated reliability throughout diagnosis and treatment [10]. Studies indicate that inflammation and cholestasis promote the progression from intrahepatic bile duct stones to ICC. Furthermore, elevated levels of GGT and ALP are associated with tumor invasiveness and the degree of biliary obstruction [11]. The levels of ALP, GGT and CA 19–9 in patients with bile duct cancer were significantly higher than those in patients with liver abscess [7].

Blood and bile cultures in this patient both identified *Escherichia coli* . Notably, ESBL‐producing Enterobacteriaceae‐induced liver abscesses frequently occur in individuals with biliary tract disorders or malignancy history [12]. Research indicates that *E. coli* may promote ICC proliferation and chemoresistance by inducing circGLIS3‐mediated stress granule formation, thereby activating the NF‐κB pathway [1]. Furthermore, gallstones show a significant association with ICC risk (OR = 6.94, 95% CI: 5.64–8.54). Most ICC risk factors (such as biliary calculi, chronic inflammation) trigger cholestasis and intracellular pathway activation, fostering reactive cellular proliferation and genetic mutations that ultimately drive cholangiocarcinogenesis [13].

## 5 Conclusion

ICC is characterized by insidious onset and rapid progression, often with early perineural and vascular invasion, while lacking typical clinical symptoms. Therefore, for patients presenting with liver abscess‐like symptoms (such as fever, hepatic pain) as the initial manifest. If imaging reveals features such as multiple bile duct stones, biliary dilatation, or duct wall thickening, a high suspicion for ICC is warranted. In this situation, jointly detecting GGT and ALP in liver function, as well as tumor marker CA19‐9, and combining the delayed enhancement patterns of contrast‐enhanced CT/MRI and DWI‐ADC signal features, can significantly reduce the risk of misdiagnosis between ICC and liver abscess.

## Author Contributions

**Chunxiao Song:** conceptualization, writing – original draft, writing – review and editing. **Yang Xu:** conceptualization, writing – original draft, writing – review and editing. **Yuanyuan Zhang:** conceptualization, writing – original draft, writing – review and editing. **Runfeng Miao:** conceptualization, writing – original draft, writing – review and editing.

## Funding

The authors have nothing to report.

## Consent

Written informed consent was obtained from the patient to publish this report in accordance with the journal's patient consent policy.

## Conflicts of Interest

The authors declare no conflicts of interest.

## Data Availability

Data sharing not applicable to this article as no datasets were generated or analysed during the current study.
